# *catena*-Poly[[[diaqua­lanthanum(III)]-tetra­kis[μ-*N*-(4-acetamido­phenyl­sulfon­yl)­glycinato]-[diaqua­lanthanum(III)]-bis­[μ-*N*-(4-acetamido­phenyl­sulfon­yl)­glycinato]] 4,4′-bipyridine disolvate tetra­deca­hydrate]

**DOI:** 10.1107/S1600536808031450

**Published:** 2008-10-04

**Authors:** Jian-Ge Wang, Jian-Hua Qin

**Affiliations:** a College of Chemistry and Chemical Engineering, Luoyang Normal University, Luoyang 471022, People’s Republic of China

## Abstract

In the title compound, {[La_2_(C_10_H_11_N_2_O_5_S)_6_(H_2_O)_4_]·2C_10_H_8_N_2_·14H_2_O}_*n*_, the La^III^ ions are in a slightly distorted bicapped trigonal prismatic geometry, and are linked by six carboxyl­ate groups in a *syn*–*syn* bidentate bridging fashion to form a one-dimensional inorganic–organic alternating linear chain. These polymeric chains generate microchannels extending along [100], and these cavities are occupied by discrete tetra­deca­meric water clusters, which inter­act with their surroundings and finally furnish the three-dimensional supra­molecular network *via* 15 O—H⋯O, one O—H⋯S, two O—H⋯N and six N—H⋯O classical hydrogen bonds. 4,4-Bipyridine acts as an inserting component and hydrogen-bond acceptor, and it is a nonplanar mol­ecule with a dihedral angle of 33.12 (13)° between the pyridine rings. Owing to the numerous classical hydrogen bonds, the observed weak inter­molecular C—H⋯O, C—H⋯π and π–π stacking inter­actions can be neglected with regard to stabilizing the network.

## Related literature

For the structure of a related complex, see: Hu *et al.* (2007 ▶). For other related literature on lanthanides, see: Guo *et al.* (2005 ▶); Pan *et al.* (2003 ▶); Zhao *et al.* (2004 ▶); Zheng *et al.* (2004 ▶).
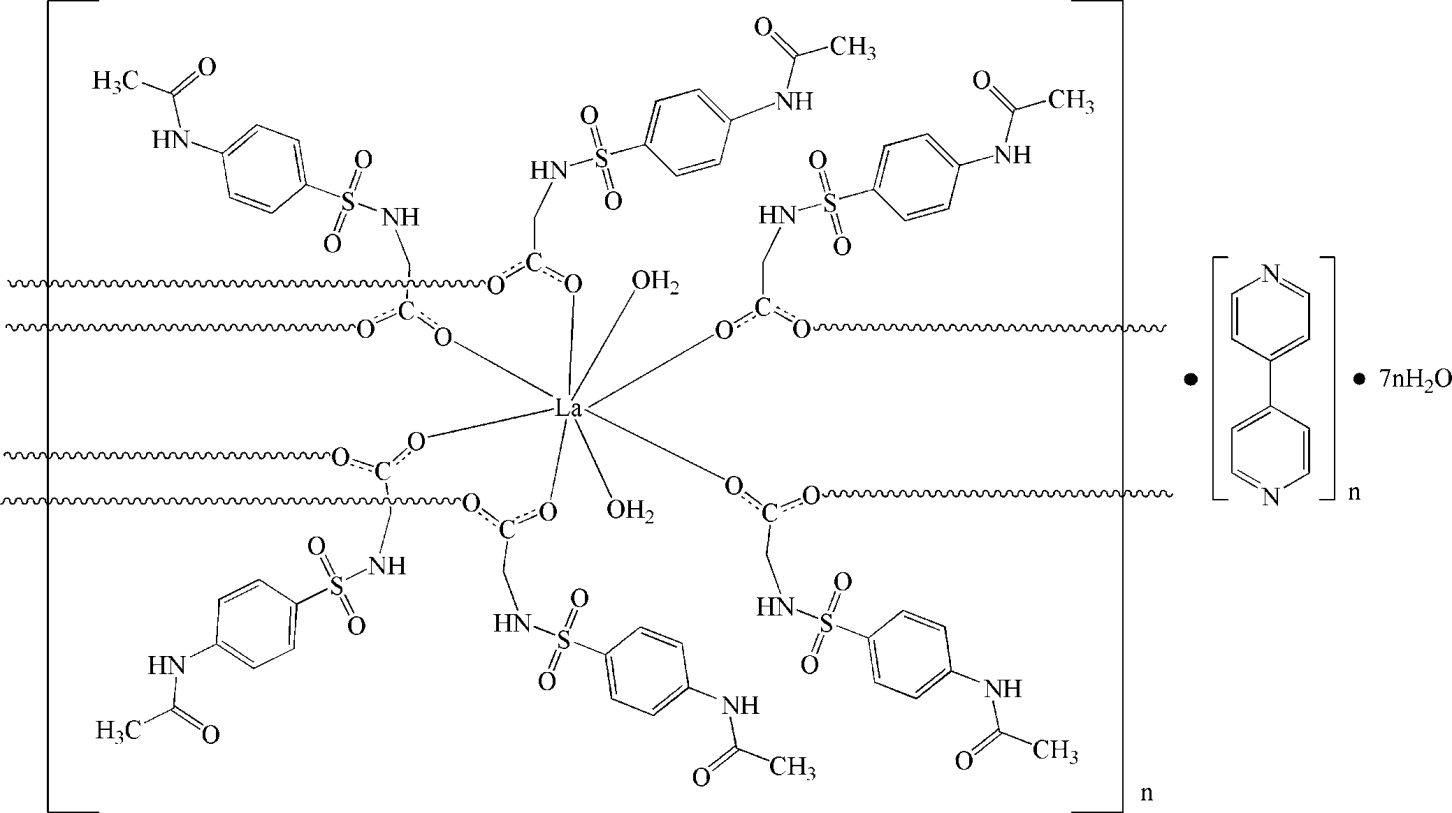


## Experimental

### 

#### Crystal data

[La_2_(C_10_H_11_N_2_O_5_S)_6_(H_2_O)_4_]·2C_10_H_8_N_2_·14H_2_O
*M* *_r_* = 2542.08
Triclinic, 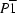

*a* = 9.6379 (8) Å
*b* = 16.9589 (13) Å
*c* = 17.6005 (14) Å
α = 99.971 (1)°
β = 105.758 (1)°
γ = 93.692 (1)°
*V* = 2707.9 (4) Å^3^
*Z* = 1
Mo *K*α radiation
μ = 0.99 mm^−1^
*T* = 291 (2) K
0.25 × 0.13 × 0.08 mm

#### Data collection

Bruker SMART CCD area-detector diffractometer
Absorption correction: multi-scan (*SADABS*; Bruker, 1997 ▶) *T*_min_ = 0.790, *T*_max_ = 0.925
20875 measured reflections
10013 independent reflections
8632 reflections with *I* > 2σ(*I*)
*R*_int_ = 0.028

#### Refinement

*R*[*F* ^2^ > 2σ(*F* ^2^)] = 0.032
*wR*(*F* ^2^) = 0.072
*S* = 1.02
10013 reflections
688 parameters
H-atom parameters constrained
Δρ_max_ = 0.39 e Å^−3^
Δρ_min_ = −0.48 e Å^−3^

### 

Data collection: *SMART* (Bruker, 1997 ▶); cell refinement: *SAINT* (Bruker, 1997 ▶); data reduction: *SAINT*; program(s) used to solve structure: *SHELXS97* (Sheldrick, 2008 ▶); program(s) used to refine structure: *SHELXL97* (Sheldrick, 2008 ▶); molecular graphics: *SHELXTL* (Sheldrick, 2008 ▶); software used to prepare material for publication: *SHELXTL*.

## Supplementary Material

Crystal structure: contains datablocks global, I. DOI: 10.1107/S1600536808031450/si2111sup1.cif

Structure factors: contains datablocks I. DOI: 10.1107/S1600536808031450/si2111Isup2.hkl

Additional supplementary materials:  crystallographic information; 3D view; checkCIF report

## Figures and Tables

**Table 1:** Hydrogen-bond geometry (Å, °)

*D*—H⋯*A*	*D*—H	H⋯*A*	*D*⋯*A*	*D*—H⋯*A*
O1—H1*W*⋯O23^i^	0.82	2.11	2.872 (3)	153
O1—H2*W*⋯O2^ii^	0.83	1.99	2.818 (3)	179
O2—H3*W*⋯N2	0.84	2.00	2.827 (4)	171
O2—H4*W*⋯O4^ii^	0.83	1.98	2.744 (3)	154
O3—H5*W*⋯O9^iii^	0.85	1.98	2.801 (4)	162
O3—H6*W*⋯O14^iv^	0.84	1.94	2.772 (3)	175
O4—H7*W*⋯N1^v^	0.83	1.99	2.781 (4)	158
O4—H8*W*⋯O12^vi^	0.82	2.41	3.166 (3)	154
O4—H8*W*⋯S1^vi^	0.82	2.94	3.711 (3)	156
O5—H9*W*⋯O4	0.84	2.04	2.865 (4)	167
O5—H10*W*⋯O11	0.83	2.03	2.844 (4)	168
O6—H11*W*⋯O5	0.84	1.91	2.716 (4)	160
O6—H12*W*⋯O18	0.84	2.01	2.805 (3)	158
O7—H13*W*⋯O13	0.83	2.12	2.914 (4)	160
O7—H14*W*⋯O6	0.84	2.00	2.810 (4)	165
O8—H15*W*⋯O19^v^	0.83	2.00	2.722 (4)	145
O8—H16*W*⋯O7	0.91	1.88	2.708 (4)	151
O9—H17*W*⋯O8	0.83	2.00	2.751 (4)	151
N3—H3⋯O6^vii^	0.86	2.15	3.007 (4)	171
N4—H4⋯O16^viii^	0.85	2.30	3.151 (3)	173
N5—H5⋯O3^vii^	0.86	2.06	2.921 (4)	177
N6—H6⋯O20	0.86	2.19	3.040 (3)	169
N7—H7⋯O8^ix^	0.86	2.02	2.878 (4)	172
N8—H8⋯O17	0.86	2.33	2.974 (3)	131

Symmetry codes: (i) 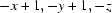; (ii) 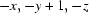; (iii) 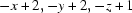; (iv) 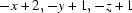; (v) 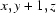; (vi) 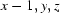; (vii) 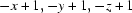; (viii) 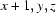; (ix) 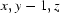.

**Fig. 1.**
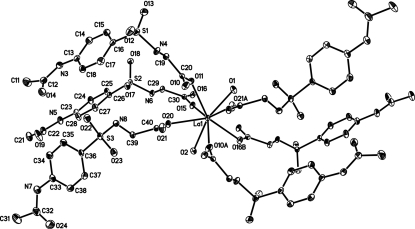
A view of the title compound, H atoms, the 4,4'-bipyridine and the hydrate water molecules are omitted for clarity. The symmetry-related three ligands are unlabelled except for O10A, O21A, O16B. Symmetry code: A = 1 - x, 1 - y, -z, B = -x, 1 - y, -z.

**Fig. 2.**
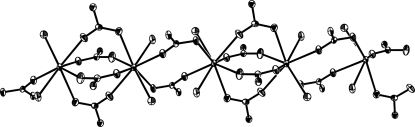
A view of the chain structure of (I).

## supplementary crystallographic information

### Comment

Organic carboxylates or N-donor ligands have been widely used in construction of coordination polymers containing transition metals. Meanwhile, in contrast to the well investigated transition metal system, the lanthanide coordination polymers have been less studied (Pan *et al.*, 2003; Zhao *et al.*, 2004; Guo *et al.*, 2005). Whereas, lanthanide ions, with their high and variable coordination numbers, flexible coordination environments and luminescence properties, provide unique opportunities for discovery of unusual network topologies, biochemical sensors and fluoroimmunoassays (Hu *et al.*, 2007; Zheng *et al.*, 2004).

In the title compound, the asymmetric structure unit consists of one La^III^ ion, three *N*—*p*-acetamidobenzenesulfonyl-glycine acid(abglyH_2_) ligands, two coordinated water molecules, one uncoordinated 4,4-bipy molecule and seven lattice water molecules. The coordination geometry of the La ion shows a slightly distorted bicapped trigonal prism (Fig. 1). The alternation of two and four bridging abglyH^-^ ligands between adjacent La centers gives one-dimensional inorganic-organic alternating linear chains (Fig. 2), which are further connected to generate a three-dimensional supramolecular structure, by O—H···O, O—H···N, and N—H···O hydrogen bonds (Table 1). 4,4-bipyridine acts as an inserting component that has a subtle effect on the structural characteristics by self-assembled control, not as an excellent rodlike bifunctional bridging ligand as in the other multidimensional mixed-ligand coordination systems, which may result from the nature of lanthanide ions having a strong affinity to oxygen atom. 4,4-bipyridine is a non-planar molecule and the dihedral angle is 33.12 (13)°.

### Experimental

The mixture of La(NO_3_)_3_(0.2 mmol) and *N*-*p*-acetamidobenzenesulfonyl-glycine acid (abglyH_2_)(0.6 mmol), was stirred into 15 ml aqueous solution. Then the pH was adjusted to 5 or so with 1 *M* NaOH. And then 3 ml ethanol solution of 4, 4'-bipyridine (0.2 mmol) was added. The reaction mixture was heated on a water bath for 10 h at 343 K, and then filtered. Colorless crystals were produced after 20 days.

### Refinement

Water H atoms were located in a difference Fourier and allowed to ride in the range 0.80 - 0.91 Å with *U*_iso_(H) = 1.5 *U*_eq_(O). Other H atoms were positioned geometrically and refined as riding atoms, with C—H = 0.93 Å (CH) and *U*ĩso~(H) = 1.2Ueq(C), with C—H = 0.97 Å (CH2) and *U*ĩso~(H) = 1.2Ueq(C), with C—H = 0.96 Å(CH3) and *U*ĩso~(H) = 1.5Ueq(C) and with N—H = 0.86 Å (NH) and *U*ĩso~(H) = 1.2Ueq(N).

### Crystal data

**Table:**

[La_2_(C_10_H_11_N_2_O_5_S)_6_(H_2_O)_4_]·2C_10_H_8_N_2_·14H_2_O	*Z* = 1
*M**_r_* = 2542.08	*F*(000) = 1304
Triclinic, *P*1	*D*_x_ = 1.559 Mg m^−^^3^
*a* = 9.6379 (8) Å	Mo *K*α radiation, λ = 0.71073 Å
*b* = 16.9589 (13) Å	Cell parameters from 6389 reflections
*c* = 17.6005 (14) Å	θ = 2.5–26.6°
α = 99.971 (1)°	µ = 0.99 mm^−^^1^
β = 105.758 (1)°	*T* = 291 K
γ = 93.692 (1)°	Block, colorless
*V* = 2707.9 (4) Å^3^	0.25 × 0.13 × 0.08 mm

### Data collection

**Table:**

Bruker SMART CCD area-detector diffractometer	10013 independent reflections
Radiation source: fine-focus sealed tube	8632 reflections with *I* > 2σ(*I*)
graphite	*R*_int_ = 0.028
φ and ω scans	θ_max_ = 25.5°, θ_min_ = 2.4°
Absorption correction: multi-scan (SADABS; Bruker, 1997)	*h* = −11→11
*T*_min_ = 0.790, *T*_max_ = 0.925	*k* = −20→20
20875 measured reflections	*l* = −21→21

### Refinement

**Table:**

Refinement on *F*^2^	Primary atom site location: structure-invariant direct methods
Least-squares matrix: full	Secondary atom site location: difference Fourier map
*R*[*F*^2^ > 2σ(*F*^2^)] = 0.032	Hydrogen site location: inferred from neighbouring sites
*wR*(*F*^2^) = 0.072	H-atom parameters constrained
*S* = 1.02	*w* = 1/[σ^2^(*F*_o_^2^) + (0.0313*P*)^2^ + 0.7554*P*] where *P* = (*F*_o_^2^ + 2*F*_c_^2^)/3
10013 reflections	(Δ/σ)_max_ = 0.001
688 parameters	Δρ_max_ = 0.39 e Å^−^^3^
0 restraints	Δρ_min_ = −0.48 e Å^−^^3^

### Special details

**Table:**

Geometry. All e.s.d.'s (except the e.s.d. in the dihedral angle between two l.s. planes)are estimated using the full covariance matrix. The cell e.s.d.'s are takeninto account individually in the estimation of e.s.d.'s in distances, anglesand torsion angles; correlations between e.s.d.'s in cell parameters are onlyused when they are defined by crystal symmetry. An approximate (isotropic)treatment of cell e.s.d.'s is used for estimating e.s.d.'s involving l.s. planes.
Refinement. Refinement of *F*^2^ against ALL reflections. The weighted *R*-factor *wR* and goodness of fit *S* are based on *F*^2^, conventional *R*-factors *R* are based on *F*, with *F* set to zero for negative *F*^2^. The threshold expression of *F*^2^ > σ(*F*^2^) is used only for calculating *R*-factors(gt) *etc*. and is not relevant to the choice of reflections for refinement. *R*-factors based on *F*^2^ are statistically about twice as large as those based on *F*, and *R*- factors based on ALL data will be even larger.

### Fractional atomic coordinates and isotropic or equivalent isotropic displacement parameters (Å^2^)

**Table:**

	*x*	*y*	*z*	*U*_iso_*/*U*_eq_	
La1	0.258177 (16)	0.499734 (10)	−0.006109 (9)	0.02304 (6)	
S1	0.89294 (8)	0.61909 (5)	0.34921 (5)	0.03773 (19)	
S2	0.40219 (7)	0.42159 (4)	0.29358 (4)	0.02678 (16)	
S3	0.79434 (8)	0.25355 (4)	0.15572 (5)	0.03198 (17)	
O1	0.1618 (2)	0.63523 (12)	0.02011 (13)	0.0401 (5)	
H1W	0.1905	0.6766	0.0076	0.060*	
H2W	0.0832	0.6385	0.0304	0.060*	
O2	0.1060 (2)	0.35582 (12)	−0.05495 (13)	0.0386 (5)	
H3W	0.1297	0.3181	−0.0310	0.058*	
H4W	0.0889	0.3404	−0.1041	0.058*	
O3	0.9777 (3)	0.89637 (17)	0.55106 (19)	0.0810 (9)	
H5W	1.0301	0.9414	0.5706	0.121*	
H6W	1.0277	0.8587	0.5598	0.121*	
O4	0.0218 (3)	0.72632 (16)	0.20987 (16)	0.0789 (9)	
H7W	0.0659	0.7723	0.2191	0.118*	
H8W	0.0159	0.7125	0.2516	0.118*	
O5	0.2932 (4)	0.6603 (2)	0.24267 (18)	0.1020 (11)	
H9W	0.2167	0.6808	0.2264	0.153*	
H10W	0.3196	0.6362	0.2049	0.153*	
O6	0.4132 (3)	0.64998 (15)	0.39867 (17)	0.0699 (8)	
H11W	0.3585	0.6546	0.3543	0.105*	
H12W	0.4297	0.6019	0.3966	0.105*	
O7	0.6218 (3)	0.78156 (17)	0.4194 (2)	0.0926 (10)	
H13W	0.6996	0.7618	0.4238	0.139*	
H14W	0.5515	0.7454	0.4052	0.139*	
O8	0.6063 (3)	0.91138 (16)	0.34828 (18)	0.0782 (9)	
H15W	0.5455	0.9364	0.3641	0.117*	
H16W	0.5898	0.8604	0.3557	0.117*	
O9	0.9044 (4)	0.9414 (2)	0.3991 (2)	0.1052 (11)	
H17W	0.8183	0.9502	0.3867	0.158*	
H18W	0.9513	0.9024	0.4112	0.158*	
O10	0.6339 (2)	0.56824 (14)	0.10554 (12)	0.0466 (6)	
O11	0.4217 (2)	0.57523 (12)	0.13048 (12)	0.0365 (5)	
O12	1.0380 (2)	0.61964 (15)	0.34221 (14)	0.0547 (7)	
O13	0.8544 (3)	0.68756 (14)	0.39538 (14)	0.0525 (6)	
O14	0.8725 (3)	0.23576 (15)	0.42586 (15)	0.0578 (7)	
O15	0.1129 (2)	0.48426 (12)	0.08728 (11)	0.0336 (5)	
O16	−0.07047 (19)	0.50076 (12)	0.14005 (11)	0.0338 (5)	
O17	0.53348 (19)	0.41848 (12)	0.27017 (12)	0.0346 (5)	
O18	0.3960 (2)	0.48139 (12)	0.36059 (12)	0.0397 (5)	
O19	0.4620 (3)	0.04174 (16)	0.3733 (2)	0.0774 (9)	
O20	0.3830 (2)	0.39887 (12)	0.06651 (12)	0.0353 (5)	
O21	0.6009 (2)	0.39877 (14)	0.04656 (14)	0.0490 (6)	
O22	0.9197 (2)	0.28575 (13)	0.22222 (13)	0.0436 (5)	
O23	0.8024 (2)	0.24757 (13)	0.07503 (13)	0.0435 (5)	
O24	0.5300 (4)	−0.14674 (17)	0.0583 (2)	0.0925 (11)	
N1	0.1019 (4)	−0.1134 (2)	0.2089 (2)	0.0775 (11)	
N2	0.1528 (3)	0.22331 (19)	0.0229 (2)	0.0563 (8)	
N3	0.7608 (3)	0.33389 (16)	0.48231 (15)	0.0403 (6)	
H3	0.7066	0.3425	0.5138	0.048*	
N4	0.7902 (2)	0.60788 (15)	0.25785 (14)	0.0345 (6)	
H4	0.8214	0.5761	0.2250	0.041*	
N5	0.2777 (3)	0.11179 (16)	0.39024 (17)	0.0453 (7)	
H5	0.2003	0.1090	0.4055	0.054*	
N6	0.2788 (2)	0.43271 (14)	0.21596 (14)	0.0299 (5)	
H6	0.2968	0.4242	0.1702	0.036*	
N7	0.6052 (3)	−0.07706 (17)	0.18688 (19)	0.0569 (8)	
H7	0.6140	−0.0819	0.2357	0.068*	
N8	0.6694 (3)	0.30760 (15)	0.16910 (15)	0.0364 (6)	
H8	0.6871	0.3433	0.2127	0.044*	
C1	0.0358 (4)	−0.0486 (3)	0.2233 (2)	0.0661 (11)	
H1	−0.0241	−0.0493	0.2568	0.079*	
C2	0.0498 (4)	0.0198 (2)	0.1921 (2)	0.0566 (10)	
H2	0.0004	0.0632	0.2048	0.068*	
C3	0.1373 (4)	0.0238 (2)	0.1419 (2)	0.0543 (9)	
C4	0.2095 (5)	−0.0430 (3)	0.1278 (3)	0.0819 (15)	
H4A	0.2716	−0.0434	0.0955	0.098*	
C5	0.1892 (6)	−0.1087 (3)	0.1616 (4)	0.1008 (18)	
H5A	0.2391	−0.1525	0.1510	0.121*	
C6	0.1388 (4)	0.2322 (2)	0.0971 (2)	0.0538 (9)	
H6A	0.1310	0.2837	0.1229	0.065*	
C7	0.1351 (4)	0.1704 (2)	0.1382 (2)	0.0491 (9)	
H7A	0.1246	0.1806	0.1898	0.059*	
C8	0.1470 (4)	0.0932 (2)	0.1023 (2)	0.0478 (9)	
C9	0.1627 (5)	0.0831 (2)	0.0258 (3)	0.0688 (12)	
H9	0.1717	0.0322	−0.0009	0.083*	
C10	0.1652 (5)	0.1486 (3)	−0.0111 (3)	0.0710 (12)	
H10	0.1762	0.1399	−0.0627	0.085*	
C11	0.7557 (5)	0.2052 (2)	0.5219 (2)	0.0692 (12)	
H11A	0.6888	0.1607	0.4873	0.104*	
H11B	0.7094	0.2352	0.5575	0.104*	
H11C	0.8393	0.1853	0.5530	0.104*	
C12	0.8020 (4)	0.2592 (2)	0.4717 (2)	0.0442 (8)	
C13	0.7945 (3)	0.39958 (19)	0.44881 (17)	0.0342 (7)	
C14	0.7545 (4)	0.4732 (2)	0.47753 (18)	0.0436 (8)	
H14	0.7073	0.4773	0.5174	0.052*	
C15	0.7837 (4)	0.5404 (2)	0.44775 (18)	0.0426 (8)	
H15	0.7568	0.5897	0.4677	0.051*	
C16	0.8528 (3)	0.53434 (18)	0.38848 (17)	0.0326 (7)	
C17	0.8907 (3)	0.4608 (2)	0.35814 (19)	0.0414 (8)	
H17	0.9350	0.4566	0.3171	0.050*	
C18	0.8631 (3)	0.3936 (2)	0.38838 (19)	0.0405 (8)	
H18	0.8903	0.3444	0.3684	0.049*	
C19	0.6322 (3)	0.60124 (18)	0.24138 (17)	0.0315 (7)	
H19A	0.6044	0.6521	0.2640	0.038*	
H19B	0.6010	0.5600	0.2671	0.038*	
C20	0.5574 (3)	0.57995 (16)	0.15174 (17)	0.0278 (6)	
C21	0.2910 (4)	−0.0189 (2)	0.4262 (3)	0.0635 (11)	
H21A	0.2954	−0.0701	0.3941	0.095*	
H21B	0.1918	−0.0127	0.4242	0.095*	
H21C	0.3467	−0.0165	0.4810	0.095*	
C22	0.3518 (4)	0.0474 (2)	0.3941 (2)	0.0489 (9)	
C23	0.3099 (3)	0.18314 (18)	0.36461 (19)	0.0371 (7)	
C24	0.2253 (3)	0.24512 (19)	0.3760 (2)	0.0425 (8)	
H24	0.1507	0.2379	0.3993	0.051*	
C25	0.2512 (3)	0.31701 (19)	0.35326 (18)	0.0382 (7)	
H25	0.1944	0.3582	0.3613	0.046*	
C26	0.3621 (3)	0.32816 (17)	0.31820 (16)	0.0291 (6)	
C27	0.4451 (3)	0.26621 (19)	0.30571 (19)	0.0396 (8)	
H27	0.5189	0.2734	0.2819	0.048*	
C28	0.4193 (4)	0.1935 (2)	0.3285 (2)	0.0448 (8)	
H28	0.4749	0.1520	0.3195	0.054*	
C29	0.1371 (3)	0.45656 (18)	0.21862 (17)	0.0293 (6)	
H29A	0.1504	0.5004	0.2643	0.035*	
H29B	0.0794	0.4115	0.2267	0.035*	
C30	0.0555 (3)	0.48297 (16)	0.14311 (16)	0.0242 (6)	
C31	0.5402 (6)	−0.2210 (2)	0.1623 (3)	0.0936 (16)	
H31A	0.4702	−0.2606	0.1222	0.140*	
H31B	0.5082	−0.2087	0.2096	0.140*	
H31C	0.6322	−0.2418	0.1757	0.140*	
C32	0.5558 (4)	−0.1454 (2)	0.1296 (3)	0.0618 (11)	
C33	0.6435 (4)	0.00059 (19)	0.1758 (2)	0.0422 (8)	
C34	0.6969 (4)	0.0612 (2)	0.2442 (2)	0.0486 (9)	
H34	0.7012	0.0497	0.2945	0.058*	
C35	0.7435 (4)	0.1381 (2)	0.2387 (2)	0.0449 (8)	
H35	0.7798	0.1779	0.2849	0.054*	
C36	0.7356 (3)	0.15532 (17)	0.16363 (18)	0.0328 (7)	
C37	0.6801 (4)	0.0964 (2)	0.0957 (2)	0.0467 (8)	
H37	0.6738	0.1084	0.0454	0.056*	
C38	0.6338 (4)	0.0197 (2)	0.1014 (2)	0.0525 (9)	
H38	0.5958	−0.0196	0.0549	0.063*	
C39	0.5270 (3)	0.29862 (17)	0.11036 (17)	0.0307 (7)	
H39A	0.4526	0.2903	0.1368	0.037*	
H39B	0.5182	0.2513	0.0688	0.037*	
C40	0.5024 (3)	0.37176 (16)	0.07175 (16)	0.0266 (6)	

### Atomic displacement parameters (Å^2^)

**Table:**

	*U*^11^	*U*^22^	*U*^33^	*U*^12^	*U*^13^	*U*^23^
La1	0.01933 (8)	0.02993 (10)	0.02388 (9)	0.00613 (6)	0.00830 (6)	0.01146 (7)
S1	0.0345 (4)	0.0441 (5)	0.0298 (4)	−0.0070 (4)	0.0029 (3)	0.0090 (4)
S2	0.0236 (3)	0.0294 (4)	0.0283 (4)	0.0054 (3)	0.0055 (3)	0.0106 (3)
S3	0.0291 (4)	0.0326 (4)	0.0405 (4)	0.0096 (3)	0.0136 (3)	0.0156 (3)
O1	0.0328 (11)	0.0365 (12)	0.0628 (15)	0.0106 (9)	0.0250 (11)	0.0203 (11)
O2	0.0398 (12)	0.0346 (12)	0.0428 (13)	0.0061 (10)	0.0122 (10)	0.0104 (10)
O3	0.0721 (18)	0.0707 (19)	0.130 (3)	0.0270 (15)	0.0518 (19)	0.0552 (19)
O4	0.115 (2)	0.0629 (19)	0.0624 (18)	−0.0146 (17)	0.0333 (18)	0.0175 (15)
O5	0.098 (2)	0.146 (3)	0.068 (2)	0.041 (2)	0.0359 (19)	0.009 (2)
O6	0.091 (2)	0.0465 (16)	0.081 (2)	0.0101 (14)	0.0397 (17)	0.0114 (14)
O7	0.098 (2)	0.065 (2)	0.126 (3)	0.0141 (17)	0.040 (2)	0.0344 (19)
O8	0.099 (2)	0.0625 (18)	0.101 (2)	0.0310 (16)	0.0561 (19)	0.0379 (17)
O9	0.096 (2)	0.106 (3)	0.133 (3)	0.020 (2)	0.043 (2)	0.056 (2)
O10	0.0436 (13)	0.0706 (17)	0.0307 (12)	0.0262 (12)	0.0160 (10)	0.0081 (11)
O11	0.0286 (11)	0.0454 (13)	0.0337 (12)	0.0027 (9)	0.0060 (9)	0.0081 (10)
O12	0.0304 (12)	0.0812 (19)	0.0475 (14)	−0.0134 (12)	0.0032 (11)	0.0199 (13)
O13	0.0668 (16)	0.0419 (14)	0.0428 (14)	−0.0031 (12)	0.0101 (12)	0.0044 (11)
O14	0.0787 (18)	0.0514 (16)	0.0529 (16)	0.0204 (14)	0.0284 (14)	0.0162 (13)
O15	0.0289 (10)	0.0503 (13)	0.0288 (11)	0.0078 (9)	0.0136 (9)	0.0178 (10)
O16	0.0218 (10)	0.0515 (13)	0.0320 (11)	0.0142 (9)	0.0085 (9)	0.0141 (10)
O17	0.0224 (10)	0.0384 (12)	0.0452 (12)	0.0048 (9)	0.0084 (9)	0.0156 (10)
O18	0.0454 (13)	0.0376 (13)	0.0327 (12)	0.0072 (10)	0.0069 (10)	0.0043 (10)
O19	0.082 (2)	0.0539 (17)	0.132 (3)	0.0332 (15)	0.062 (2)	0.0535 (18)
O20	0.0316 (11)	0.0431 (13)	0.0377 (12)	0.0163 (10)	0.0119 (10)	0.0173 (10)
O21	0.0377 (12)	0.0573 (15)	0.0640 (16)	0.0024 (11)	0.0208 (12)	0.0350 (13)
O22	0.0305 (11)	0.0435 (13)	0.0537 (14)	0.0036 (10)	0.0049 (11)	0.0132 (11)
O23	0.0518 (14)	0.0434 (13)	0.0476 (14)	0.0143 (11)	0.0262 (11)	0.0197 (11)
O24	0.134 (3)	0.0467 (18)	0.070 (2)	−0.0152 (17)	−0.007 (2)	0.0095 (16)
N1	0.080 (3)	0.065 (2)	0.100 (3)	0.018 (2)	0.031 (2)	0.036 (2)
N2	0.0494 (18)	0.049 (2)	0.070 (2)	−0.0031 (15)	0.0138 (17)	0.0179 (17)
N3	0.0515 (16)	0.0413 (16)	0.0330 (15)	0.0045 (13)	0.0189 (13)	0.0102 (12)
N4	0.0294 (13)	0.0478 (16)	0.0263 (13)	0.0003 (12)	0.0070 (11)	0.0108 (12)
N5	0.0483 (16)	0.0425 (17)	0.0569 (18)	0.0110 (13)	0.0227 (14)	0.0273 (14)
N6	0.0282 (12)	0.0412 (15)	0.0268 (13)	0.0155 (11)	0.0112 (11)	0.0147 (11)
N7	0.076 (2)	0.0382 (18)	0.059 (2)	0.0015 (16)	0.0182 (17)	0.0183 (15)
N8	0.0349 (14)	0.0370 (15)	0.0355 (14)	0.0140 (12)	0.0063 (12)	0.0047 (12)
C1	0.067 (3)	0.071 (3)	0.066 (3)	0.010 (2)	0.023 (2)	0.025 (2)
C2	0.059 (2)	0.055 (2)	0.059 (2)	0.0136 (19)	0.019 (2)	0.0149 (19)
C3	0.053 (2)	0.049 (2)	0.068 (3)	0.0160 (18)	0.023 (2)	0.0154 (19)
C4	0.090 (3)	0.066 (3)	0.126 (4)	0.042 (3)	0.067 (3)	0.046 (3)
C5	0.108 (4)	0.071 (3)	0.166 (6)	0.050 (3)	0.078 (4)	0.061 (4)
C6	0.048 (2)	0.043 (2)	0.066 (3)	0.0040 (17)	0.0122 (19)	0.0064 (19)
C7	0.045 (2)	0.050 (2)	0.051 (2)	0.0071 (17)	0.0121 (17)	0.0077 (18)
C8	0.045 (2)	0.043 (2)	0.057 (2)	0.0082 (16)	0.0160 (18)	0.0119 (18)
C9	0.091 (3)	0.048 (2)	0.076 (3)	0.013 (2)	0.040 (3)	0.009 (2)
C10	0.094 (3)	0.062 (3)	0.062 (3)	0.000 (2)	0.033 (2)	0.010 (2)
C11	0.098 (3)	0.050 (2)	0.073 (3)	0.005 (2)	0.040 (3)	0.023 (2)
C12	0.052 (2)	0.040 (2)	0.0368 (19)	0.0004 (16)	0.0081 (17)	0.0077 (15)
C13	0.0336 (16)	0.0413 (19)	0.0251 (15)	0.0006 (14)	0.0034 (13)	0.0088 (14)
C14	0.059 (2)	0.049 (2)	0.0317 (17)	0.0097 (17)	0.0246 (16)	0.0124 (15)
C15	0.056 (2)	0.042 (2)	0.0336 (18)	0.0098 (16)	0.0166 (16)	0.0080 (15)
C16	0.0313 (16)	0.0404 (18)	0.0241 (15)	0.0005 (14)	0.0040 (13)	0.0085 (13)
C17	0.0400 (18)	0.054 (2)	0.0367 (18)	0.0067 (16)	0.0195 (15)	0.0121 (16)
C18	0.0423 (18)	0.043 (2)	0.0413 (19)	0.0145 (15)	0.0167 (16)	0.0116 (15)
C19	0.0300 (15)	0.0376 (17)	0.0289 (16)	0.0028 (13)	0.0102 (13)	0.0100 (13)
C20	0.0350 (16)	0.0223 (15)	0.0267 (15)	0.0062 (12)	0.0088 (13)	0.0056 (12)
C21	0.073 (3)	0.048 (2)	0.083 (3)	0.012 (2)	0.028 (2)	0.039 (2)
C22	0.054 (2)	0.043 (2)	0.057 (2)	0.0108 (18)	0.0187 (19)	0.0239 (17)
C23	0.0407 (18)	0.0351 (18)	0.0403 (18)	0.0054 (14)	0.0119 (15)	0.0197 (15)
C24	0.0434 (18)	0.045 (2)	0.053 (2)	0.0128 (16)	0.0244 (17)	0.0258 (17)
C25	0.0384 (17)	0.0415 (19)	0.0447 (19)	0.0167 (15)	0.0184 (15)	0.0205 (15)
C26	0.0322 (15)	0.0302 (16)	0.0269 (15)	0.0069 (13)	0.0062 (13)	0.0132 (13)
C27	0.0406 (18)	0.0427 (19)	0.048 (2)	0.0134 (15)	0.0233 (16)	0.0213 (16)
C28	0.050 (2)	0.0386 (19)	0.059 (2)	0.0211 (16)	0.0268 (18)	0.0228 (17)
C29	0.0225 (14)	0.0414 (18)	0.0305 (16)	0.0090 (13)	0.0114 (12)	0.0163 (13)
C30	0.0197 (13)	0.0260 (15)	0.0277 (15)	0.0002 (11)	0.0071 (12)	0.0075 (12)
C31	0.130 (4)	0.036 (2)	0.116 (4)	−0.001 (3)	0.032 (4)	0.027 (3)
C32	0.065 (3)	0.037 (2)	0.076 (3)	−0.0011 (19)	0.006 (2)	0.017 (2)
C33	0.0458 (19)	0.0318 (18)	0.052 (2)	0.0057 (15)	0.0139 (17)	0.0165 (16)
C34	0.068 (2)	0.042 (2)	0.043 (2)	0.0083 (18)	0.0227 (18)	0.0171 (17)
C35	0.063 (2)	0.0364 (19)	0.0403 (19)	0.0087 (16)	0.0196 (17)	0.0120 (15)
C36	0.0364 (16)	0.0282 (16)	0.0378 (17)	0.0121 (13)	0.0118 (14)	0.0126 (14)
C37	0.064 (2)	0.039 (2)	0.0360 (19)	0.0074 (17)	0.0098 (17)	0.0128 (16)
C38	0.076 (3)	0.036 (2)	0.039 (2)	0.0008 (18)	0.0058 (18)	0.0078 (16)
C39	0.0269 (15)	0.0316 (17)	0.0360 (17)	0.0065 (13)	0.0090 (13)	0.0118 (13)
C40	0.0263 (15)	0.0295 (16)	0.0261 (15)	0.0028 (12)	0.0084 (12)	0.0096 (12)

### Geometric parameters (Å, °)

**Table:**

La1—O10^i^	2.433 (2)	C1—C2	1.381 (5)
La1—O21^i^	2.442 (2)	C1—H1	0.9300
La1—O15	2.4662 (18)	C2—C3	1.383 (5)
La1—O20	2.4887 (19)	C2—H2	0.9300
La1—O1	2.549 (2)	C3—C4	1.388 (5)
La1—O16^ii^	2.5505 (18)	C3—C8	1.479 (5)
La1—O11	2.553 (2)	C4—C5	1.378 (6)
La1—O2	2.641 (2)	C4—H4A	0.9300
S1—O13	1.428 (2)	C5—H5A	0.9300
S1—O12	1.436 (2)	C6—C7	1.377 (5)
S1—N4	1.615 (2)	C6—H6A	0.9300
S1—C16	1.764 (3)	C7—C8	1.378 (5)
S2—O18	1.434 (2)	C7—H7A	0.9300
S2—O17	1.4345 (19)	C8—C9	1.377 (5)
S2—N6	1.598 (2)	C9—C10	1.383 (5)
S2—C26	1.760 (3)	C9—H9	0.9300
S3—O23	1.430 (2)	C10—H10	0.9300
S3—O22	1.433 (2)	C11—C12	1.502 (5)
S3—N8	1.602 (2)	C11—H11A	0.9600
S3—C36	1.765 (3)	C11—H11B	0.9600
O1—H1W	0.8232	C11—H11C	0.9600
O1—H2W	0.8290	C13—C14	1.383 (4)
O2—H3W	0.8361	C13—C18	1.390 (4)
O2—H4W	0.8265	C14—C15	1.379 (4)
O3—H5W	0.8497	C14—H14	0.9300
O3—H6W	0.8351	C15—C16	1.375 (4)
O4—H7W	0.8315	C15—H15	0.9300
O4—H8W	0.8231	C16—C17	1.381 (4)
O5—H9W	0.8356	C17—C18	1.379 (4)
O5—H10W	0.8273	C17—H17	0.9300
O6—H11W	0.8353	C18—H18	0.9300
O6—H12W	0.8372	C19—C20	1.513 (4)
O7—H13W	0.8312	C19—H19A	0.9700
O7—H14W	0.8362	C19—H19B	0.9700
O8—H15W	0.8261	C21—C22	1.498 (4)
O8—H16W	0.9075	C21—H21A	0.9600
O9—H17W	0.8282	C21—H21B	0.9600
O9—H18W	0.8497	C21—H21C	0.9600
O10—C20	1.238 (3)	C23—C28	1.388 (4)
O10—La1^i^	2.433 (2)	C23—C24	1.391 (4)
O11—C20	1.252 (3)	C24—C25	1.376 (4)
O14—C12	1.221 (4)	C24—H24	0.9300
O15—C30	1.254 (3)	C25—C26	1.390 (4)
O16—C30	1.259 (3)	C25—H25	0.9300
O16—La1^ii^	2.5505 (18)	C26—C27	1.385 (4)
O19—C22	1.217 (4)	C27—C28	1.388 (4)
O20—C40	1.253 (3)	C27—H27	0.9300
O21—C40	1.245 (3)	C28—H28	0.9300
O21—La1^i^	2.442 (2)	C29—C30	1.506 (4)
O24—C32	1.207 (5)	C29—H29A	0.9700
N1—C1	1.327 (5)	C29—H29B	0.9700
N1—C5	1.343 (6)	C31—C32	1.507 (5)
N2—C10	1.332 (5)	C31—H31A	0.9600
N2—C6	1.333 (5)	C31—H31B	0.9600
N3—C12	1.350 (4)	C31—H31C	0.9600
N3—C13	1.407 (4)	C33—C38	1.382 (4)
N3—H3	0.8600	C33—C34	1.392 (5)
N4—C19	1.463 (3)	C34—C35	1.378 (4)
N4—H4	0.8505	C34—H34	0.9300
N5—C22	1.345 (4)	C35—C36	1.386 (4)
N5—C23	1.407 (4)	C35—H35	0.9300
N5—H5	0.8600	C36—C37	1.372 (4)
N6—C29	1.459 (3)	C37—C38	1.377 (4)
N6—H6	0.8600	C37—H37	0.9300
N7—C32	1.360 (5)	C38—H38	0.9300
N7—C33	1.405 (4)	C39—C40	1.516 (4)
N7—H7	0.8600	C39—H39A	0.9700
N8—C39	1.457 (3)	C39—H39B	0.9700
N8—H8	0.8600		
			
O10^i^—La1—O21^i^	71.49 (8)	N2—C10—C9	123.8 (4)
O10^i^—La1—O15	146.41 (8)	N2—C10—H10	118.1
O21^i^—La1—O15	141.98 (8)	C9—C10—H10	118.1
O10^i^—La1—O20	79.17 (7)	C12—C11—H11A	109.5
O21^i^—La1—O20	119.96 (7)	C12—C11—H11B	109.5
O15—La1—O20	78.48 (6)	H11A—C11—H11B	109.5
O10^i^—La1—O1	135.58 (7)	C12—C11—H11C	109.5
O21^i^—La1—O1	70.64 (7)	H11A—C11—H11C	109.5
O15—La1—O1	75.22 (6)	H11B—C11—H11C	109.5
O20—La1—O1	141.13 (7)	O14—C12—N3	123.9 (3)
O10^i^—La1—O16^ii^	76.46 (6)	O14—C12—C11	121.3 (3)
O21^i^—La1—O16^ii^	84.20 (7)	N3—C12—C11	114.8 (3)
O15—La1—O16^ii^	104.41 (6)	C14—C13—C18	119.3 (3)
O20—La1—O16^ii^	137.39 (7)	C14—C13—N3	117.3 (3)
O1—La1—O16^ii^	77.47 (7)	C18—C13—N3	123.5 (3)
O10^i^—La1—O11	119.68 (7)	C15—C14—C13	120.8 (3)
O21^i^—La1—O11	78.85 (7)	C15—C14—H14	119.6
O15—La1—O11	76.15 (6)	C13—C14—H14	119.6
O20—La1—O11	72.14 (7)	C16—C15—C14	119.8 (3)
O1—La1—O11	74.11 (7)	C16—C15—H15	120.1
O16^ii^—La1—O11	150.42 (7)	C14—C15—H15	120.1
O10^i^—La1—O2	76.66 (7)	C15—C16—C17	120.0 (3)
O21^i^—La1—O2	143.98 (7)	C15—C16—S1	121.1 (2)
O15—La1—O2	71.94 (6)	C17—C16—S1	118.9 (2)
O20—La1—O2	68.44 (6)	C18—C17—C16	120.4 (3)
O1—La1—O2	127.18 (6)	C18—C17—H17	119.8
O16^ii^—La1—O2	72.22 (6)	C16—C17—H17	119.8
O11—La1—O2	133.04 (6)	C17—C18—C13	119.7 (3)
O13—S1—O12	119.51 (15)	C17—C18—H18	120.1
O13—S1—N4	107.25 (14)	C13—C18—H18	120.1
O12—S1—N4	104.86 (13)	N4—C19—C20	111.1 (2)
O13—S1—C16	107.34 (14)	N4—C19—H19A	109.4
O12—S1—C16	108.83 (14)	C20—C19—H19A	109.4
N4—S1—C16	108.67 (13)	N4—C19—H19B	109.4
O18—S2—O17	119.95 (13)	C20—C19—H19B	109.4
O18—S2—N6	108.07 (12)	H19A—C19—H19B	108.0
O17—S2—N6	106.06 (12)	O10—C20—O11	125.3 (3)
O18—S2—C26	105.53 (13)	O10—C20—C19	118.1 (2)
O17—S2—C26	107.46 (12)	O11—C20—C19	116.6 (2)
N6—S2—C26	109.53 (13)	C22—C21—H21A	109.5
O23—S3—O22	119.92 (13)	C22—C21—H21B	109.5
O23—S3—N8	109.74 (13)	H21A—C21—H21B	109.5
O22—S3—N8	105.41 (13)	C22—C21—H21C	109.5
O23—S3—C36	106.02 (14)	H21A—C21—H21C	109.5
O22—S3—C36	108.98 (13)	H21B—C21—H21C	109.5
N8—S3—C36	106.02 (13)	O19—C22—N5	123.4 (3)
La1—O1—H1W	124.8	O19—C22—C21	120.7 (3)
La1—O1—H2W	121.2	N5—C22—C21	115.9 (3)
H1W—O1—H2W	112.0	C28—C23—C24	119.6 (3)
La1—O2—H3W	121.2	C28—C23—N5	123.3 (3)
La1—O2—H4W	111.1	C24—C23—N5	117.1 (3)
H3W—O2—H4W	110.5	C25—C24—C23	120.5 (3)
H5W—O3—H6W	110.0	C25—C24—H24	119.7
H7W—O4—H8W	111.8	C23—C24—H24	119.7
H9W—O5—H10W	111.9	C24—C25—C26	120.1 (3)
H11W—O6—H12W	109.2	C24—C25—H25	120.0
H13W—O7—H14W	110.9	C26—C25—H25	120.0
H15W—O8—H16W	105.5	C27—C26—C25	119.5 (3)
H17W—O9—H18W	137.0	C27—C26—S2	120.0 (2)
C20—O10—La1^i^	160.8 (2)	C25—C26—S2	120.5 (2)
C20—O11—La1	124.64 (18)	C26—C27—C28	120.6 (3)
C30—O15—La1	171.01 (18)	C26—C27—H27	119.7
C30—O16—La1^ii^	121.43 (17)	C28—C27—H27	119.7
C40—O20—La1	133.60 (18)	C23—C28—C27	119.7 (3)
C40—O21—La1^i^	156.3 (2)	C23—C28—H28	120.2
C1—N1—C5	115.3 (4)	C27—C28—H28	120.2
C10—N2—C6	115.4 (3)	N6—C29—C30	113.0 (2)
C12—N3—C13	128.7 (3)	N6—C29—H29A	109.0
C12—N3—H3	115.6	C30—C29—H29A	109.0
C13—N3—H3	115.6	N6—C29—H29B	109.0
C19—N4—S1	120.13 (19)	C30—C29—H29B	109.0
C19—N4—H4	115.4	H29A—C29—H29B	107.8
S1—N4—H4	111.8	O15—C30—O16	124.1 (2)
C22—N5—C23	128.6 (3)	O15—C30—C29	119.7 (2)
C22—N5—H5	115.7	O16—C30—C29	116.1 (2)
C23—N5—H5	115.7	C32—C31—H31A	109.5
C29—N6—S2	123.50 (18)	C32—C31—H31B	109.5
C29—N6—H6	118.3	H31A—C31—H31B	109.5
S2—N6—H6	118.3	C32—C31—H31C	109.5
C32—N7—C33	127.8 (3)	H31A—C31—H31C	109.5
C32—N7—H7	116.1	H31B—C31—H31C	109.5
C33—N7—H7	116.1	O24—C32—N7	123.4 (4)
C39—N8—S3	122.6 (2)	O24—C32—C31	122.0 (4)
C39—N8—H8	118.7	N7—C32—C31	114.6 (4)
S3—N8—H8	118.7	C38—C33—C34	118.4 (3)
N1—C1—C2	124.5 (4)	C38—C33—N7	123.9 (3)
N1—C1—H1	117.8	C34—C33—N7	117.7 (3)
C2—C1—H1	117.8	C35—C34—C33	121.2 (3)
C1—C2—C3	120.1 (4)	C35—C34—H34	119.4
C1—C2—H2	120.0	C33—C34—H34	119.4
C3—C2—H2	120.0	C34—C35—C36	119.3 (3)
C2—C3—C4	116.0 (4)	C34—C35—H35	120.4
C2—C3—C8	121.9 (3)	C36—C35—H35	120.4
C4—C3—C8	122.0 (4)	C37—C36—C35	119.9 (3)
C5—C4—C3	120.0 (4)	C37—C36—S3	120.3 (2)
C5—C4—H4A	120.0	C35—C36—S3	119.8 (2)
C3—C4—H4A	120.0	C36—C37—C38	120.7 (3)
N1—C5—C4	124.1 (4)	C36—C37—H37	119.7
N1—C5—H5A	117.9	C38—C37—H37	119.7
C4—C5—H5A	117.9	C37—C38—C33	120.5 (3)
N2—C6—C7	124.5 (3)	C37—C38—H38	119.8
N2—C6—H6A	117.7	C33—C38—H38	119.8
C7—C6—H6A	117.7	N8—C39—C40	112.1 (2)
C6—C7—C8	119.6 (4)	N8—C39—H39A	109.2
C6—C7—H7A	120.2	C40—C39—H39A	109.2
C8—C7—H7A	120.2	N8—C39—H39B	109.2
C7—C8—C9	116.7 (3)	C40—C39—H39B	109.2
C7—C8—C3	122.0 (3)	H39A—C39—H39B	107.9
C9—C8—C3	121.2 (3)	O21—C40—O20	125.6 (3)
C8—C9—C10	120.0 (4)	O21—C40—C39	117.5 (2)
C8—C9—H9	120.0	O20—C40—C39	116.9 (2)
C10—C9—H9	120.0		
			
O10^i^—La1—O11—C20	−0.6 (2)	C14—C13—C18—C17	0.2 (5)
O21^i^—La1—O11—C20	−61.6 (2)	N3—C13—C18—C17	179.2 (3)
O15—La1—O11—C20	147.3 (2)	S1—N4—C19—C20	−172.33 (19)
O20—La1—O11—C20	65.1 (2)	La1^i^—O10—C20—O11	−65.3 (7)
O1—La1—O11—C20	−134.4 (2)	La1^i^—O10—C20—C19	114.3 (5)
O16^ii^—La1—O11—C20	−117.9 (2)	La1—O11—C20—O10	19.9 (4)
O2—La1—O11—C20	99.1 (2)	La1—O11—C20—C19	−159.66 (18)
O10^i^—La1—O20—C40	38.9 (2)	N4—C19—C20—O10	2.6 (4)
O21^i^—La1—O20—C40	−22.2 (3)	N4—C19—C20—O11	−177.8 (2)
O15—La1—O20—C40	−166.3 (3)	C23—N5—C22—O19	−1.3 (6)
O1—La1—O20—C40	−118.2 (2)	C23—N5—C22—C21	178.5 (3)
O16^ii^—La1—O20—C40	94.8 (3)	C22—N5—C23—C28	8.6 (5)
O11—La1—O20—C40	−87.3 (3)	C22—N5—C23—C24	−172.1 (3)
O2—La1—O20—C40	118.7 (3)	C28—C23—C24—C25	−1.3 (5)
O13—S1—N4—C19	−54.5 (3)	N5—C23—C24—C25	179.4 (3)
O12—S1—N4—C19	177.4 (2)	C23—C24—C25—C26	0.3 (5)
C16—S1—N4—C19	61.2 (3)	C24—C25—C26—C27	0.6 (5)
O18—S2—N6—C29	−35.9 (3)	C24—C25—C26—S2	−176.7 (2)
O17—S2—N6—C29	−165.7 (2)	O18—S2—C26—C27	−134.9 (2)
C26—S2—N6—C29	78.6 (2)	O17—S2—C26—C27	−5.8 (3)
O23—S3—N8—C39	44.2 (3)	N6—S2—C26—C27	108.9 (3)
O22—S3—N8—C39	174.6 (2)	O18—S2—C26—C25	42.4 (3)
C36—S3—N8—C39	−69.9 (3)	O17—S2—C26—C25	171.5 (2)
C5—N1—C1—C2	1.2 (7)	N6—S2—C26—C25	−73.7 (3)
N1—C1—C2—C3	0.1 (6)	C25—C26—C27—C28	−0.5 (5)
C1—C2—C3—C4	−1.5 (6)	S2—C26—C27—C28	176.9 (3)
C1—C2—C3—C8	175.3 (4)	C24—C23—C28—C27	1.4 (5)
C2—C3—C4—C5	1.4 (7)	N5—C23—C28—C27	−179.3 (3)
C8—C3—C4—C5	−175.4 (4)	C26—C27—C28—C23	−0.5 (5)
C1—N1—C5—C4	−1.3 (8)	S2—N6—C29—C30	165.5 (2)
C3—C4—C5—N1	0.0 (9)	La1^ii^—O16—C30—O15	19.3 (4)
C10—N2—C6—C7	−0.7 (5)	La1^ii^—O16—C30—C29	−158.89 (18)
N2—C6—C7—C8	0.3 (5)	N6—C29—C30—O15	−1.5 (4)
C6—C7—C8—C9	0.2 (5)	N6—C29—C30—O16	176.8 (2)
C6—C7—C8—C3	−177.3 (3)	C33—N7—C32—O24	−4.5 (7)
C2—C3—C8—C7	33.0 (5)	C33—N7—C32—C31	173.5 (4)
C4—C3—C8—C7	−150.4 (4)	C32—N7—C33—C38	1.1 (6)
C2—C3—C8—C9	−144.4 (4)	C32—N7—C33—C34	−177.6 (4)
C4—C3—C8—C9	32.2 (6)	C38—C33—C34—C35	−2.0 (5)
C7—C8—C9—C10	−0.3 (6)	N7—C33—C34—C35	176.8 (3)
C3—C8—C9—C10	177.2 (4)	C33—C34—C35—C36	0.7 (5)
C6—N2—C10—C9	0.6 (6)	C34—C35—C36—C37	0.8 (5)
C8—C9—C10—N2	−0.1 (7)	C34—C35—C36—S3	179.8 (3)
C13—N3—C12—O14	−3.9 (5)	O23—S3—C36—C37	−13.3 (3)
C13—N3—C12—C11	175.4 (3)	O22—S3—C36—C37	−143.7 (3)
C12—N3—C13—C14	−171.7 (3)	N8—S3—C36—C37	103.3 (3)
C12—N3—C13—C18	9.3 (5)	O23—S3—C36—C35	167.7 (2)
C18—C13—C14—C15	−0.9 (5)	O22—S3—C36—C35	37.3 (3)
N3—C13—C14—C15	−180.0 (3)	N8—S3—C36—C35	−75.7 (3)
C13—C14—C15—C16	0.4 (5)	C35—C36—C37—C38	−0.8 (5)
C14—C15—C16—C17	1.0 (5)	S3—C36—C37—C38	−179.9 (3)
C14—C15—C16—S1	−179.7 (2)	C36—C37—C38—C33	−0.5 (6)
O13—S1—C16—C15	5.6 (3)	C34—C33—C38—C37	1.9 (5)
O12—S1—C16—C15	136.2 (3)	N7—C33—C38—C37	−176.8 (3)
N4—S1—C16—C15	−110.1 (3)	S3—N8—C39—C40	−112.7 (2)
O13—S1—C16—C17	−175.0 (2)	La1^i^—O21—C40—O20	25.1 (7)
O12—S1—C16—C17	−44.4 (3)	La1^i^—O21—C40—C39	−156.4 (4)
N4—S1—C16—C17	69.3 (3)	La1—O20—C40—O21	9.6 (5)
C15—C16—C17—C18	−1.7 (5)	La1—O20—C40—C39	−168.93 (18)
S1—C16—C17—C18	178.9 (2)	N8—C39—C40—O21	47.5 (4)
C16—C17—C18—C13	1.1 (5)	N8—C39—C40—O20	−133.9 (3)

Symmetry codes: (i) −*x*+1, −*y*+1, −*z*; (ii) −*x*, −*y*+1, −*z*.

### Hydrogen-bond geometry (Å, °)

**Table:**

*D*—H···*A*	*D*—H	H···*A*	*D*···*A*	*D*—H···*A*
O1—H1W···O23^i^	0.82	2.11	2.872 (3)	153
O1—H2W···O2^ii^	0.83	1.99	2.818 (3)	179
O2—H3W···N2	0.84	2.00	2.827 (4)	171
O2—H4W···O4^ii^	0.83	1.98	2.744 (3)	154
O3—H5W···O9^iii^	0.85	1.98	2.801 (4)	162
O3—H6W···O14^iv^	0.84	1.94	2.772 (3)	175
O4—H7W···N1^v^	0.83	1.99	2.781 (4)	158
O4—H8W···O12^vi^	0.82	2.41	3.166 (3)	154
O4—H8W···S1^vi^	0.82	2.94	3.711 (3)	156
O5—H9W···O4	0.84	2.04	2.865 (4)	167
O5—H10W···O11	0.83	2.03	2.844 (4)	168
O6—H11W···O5	0.84	1.91	2.716 (4)	160
O6—H12W···O18	0.84	2.01	2.805 (3)	158
O7—H13W···O13	0.83	2.12	2.914 (4)	160
O7—H14W···O6	0.84	2.00	2.810 (4)	165
O8—H15W···O19^v^	0.83	2.00	2.722 (4)	145
O8—H16W···O7	0.91	1.88	2.708 (4)	151
O9—H17W···O8	0.83	2.00	2.751 (4)	151
N3—H3···O6^vii^	0.86	2.15	3.007 (4)	171
N4—H4···O16^viii^	0.85	2.30	3.151 (3)	173
N5—H5···O3^vii^	0.86	2.06	2.921 (4)	177
N6—H6···O20	0.86	2.19	3.040 (3)	169
N7—H7···O8^ix^	0.86	2.02	2.878 (4)	172
N8—H8···O17	0.86	2.33	2.974 (3)	131

Symmetry codes: (i) −*x*+1, −*y*+1, −*z*; (ii) −*x*, −*y*+1, −*z*; (iii) −*x*+2, −*y*+2, −*z*+1; (iv) −*x*+2, −*y*+1, −*z*+1; (v) *x*, *y*+1, *z*; (vi) *x*−1, *y*, *z*; (vii) −*x*+1, −*y*+1, −*z*+1; (viii) *x*+1, *y*, *z*; (ix) *x*, *y*−1, *z*.
